# Relative frequency and estimated minimal frequency of Lysosomal Storage Diseases in Brazil: Report from a Reference Laboratory

**DOI:** 10.1590/1678-4685-GMB-2016-0268

**Published:** 2017-03-16

**Authors:** Roberto Giugliani, Andressa Federhen, Kristiane Michelin-Tirelli, Mariluce Riegel, Maira Burin

**Affiliations:** 1 Medical Genetics Service, Hospital das Clínicas de Porto Alegre (HCPA), Porto Alegre, RS, Brazil.; 2 Department of Genetics, Universidade Federal de Rio Grande do Sul (UFRGS), Porto Alegre, RS, Brazil.; 3 Post-Graduate Program in Genetics and Molecular Biology, Universidade Federal de Rio Grande do Sul (UFRGS), Porto Alegre, RS, Brazil.; 4 Post-Graduate Program in Child and Adolescent Health, Universidade Federal de Rio Grande do Sul (UFRGS), Porto Alegre, RS, Brazil.; 5 Clinical Research Group on Medical Genetics, Hospital das Clínicas de Porto Alegre (HCPA), Porto Alegre, RS, Brazil.

**Keywords:** Lysosomal storage diseases, epidemiology, reference center, biochemical genetics, Brazil

## Abstract

Lysosomal storage diseases (LSDs) comprise a heterogeneous group of more than 50 genetic conditions of inborn errors of metabolism (IEM) caused by a defect in lysosomal function. Although there are screening tests for some of these conditions, diagnosis usually depends on specific enzyme assays, which are only available in a few laboratories around the world. A pioneer facility for the diagnosis of IEM and LSDs was established in the South of Brazil in 1982 and has served as a reference service since then. Over the past 34 years, samples from 72,797 patients were referred for investigation of IEM, and 3,211 were confirmed as having an LSD (4.41%, or 1 in 22), with 3,099 of these patients originating from Brazil. The rate of diagnosis has increased over time, in part due to the creation of diagnostic networks involving a large number of Brazilian services. These cases, referred from Brazilian regions, provide insight about the relative frequency of LSDs in the country. The large amount of data available allows for the estimation of the minimal frequency of specific LSDs in Brazil. The reported data could help to plan health care policies, as there are specific therapies available for most of the cases diagnosed.

## Introduction

Lysosomal storage diseases (LSDs) comprise a heterogeneous group of more than 50 genetic progressive conditions caused by a defect in lysosomal function (Giugliani, 2012a). LSDs have a wide range of disease manifestations, including hydrops fetalis, neurocognitive decline, dysmorphia, hepatosplenomegaly and musculoskeletal abnormalities (Kingma *et al*., 2015).

LSDs usually result from a deficiency in an enzyme involved in the degradation of macromolecules, or sometimes, from a problem in the transport of molecules across the lysosomal membrane (Futerman and van Meer, 2004). The diseases are typically classified according to the type of material that accumulates (Vellodi, 2005). Clinical features vary from mild to severe, and these conditions are not evident at birth in most cases, with features becoming apparent usually in childhood. Most cases have severe manifestations, high morbidity and shortened life spans (Giugliani, 2012a). It is clear that most LSDs are heterogeneous and have a broad continuum of clinical severity and age at presentation, making their early identification difficult and causing a significant delay between disease onset and diagnosis (Wilcox, 2004).

Although LSDs are classified as rare diseases, the frequency is significant when the group is considered as a whole, varying from one case in every 4,000 to 9,000 births across different studies (Fuller *et al*., 2006). In countries where consanguinity rates are high, an increased incidence of inherited disorders is observed and can be as high as 1 in 2,200 in Saudi Arabia (Moammar *et al*., 2010).

In a retrospective study in Australia, the incidence of LSDs as a group was calculated to be 1 in 7,700, ranging from 1 in 57,000 for Gaucher disease to as low as 1 in 4.2 million for sialidosis (Meikle *et al*., 1999). Similar rates were found in a study conducted by Poorthuis *et al*. (1999) in the Netherlands, where the combined LSD frequency was 1 in 7,100 live births, with Pompe disease being the most prevalent at 1 in 50,000 (Poorthuis *et al*., 1999).

Specific protocols for selective screening of inborn errors of metabolism (IEM) in high-risk patients were introduced by the middle of the last century in several countries. Improvements in analytical equipment and techniques for assaying metabolites have allowed the diagnosis of an increasing number of disorders (Hoffmann, 1994).

Based on the experience of developed countries, a reference laboratory for the detection of IEM was established in 1982 in Southern Brazil at the Medical Genetics Service (MGS) of Hospital de Clínicas de Porto Alegre (HCPA). Currently, this facility is one of the most comprehensive laboratories for the diagnosis of lysosomal storage diseases in Latin America. This laboratory is a well-known reference center in Brazil and has been receiving samples since 1982, not only from Brazil but also from many other countries. The aim of this study was to report the experience from this reference laboratory for LSD diagnosis, to estimate the relative frequency and minimal frequency of these diseases in Brazil, and to compare this information to the reported frequencies from other countries.

## Methods

**Figure 1:**
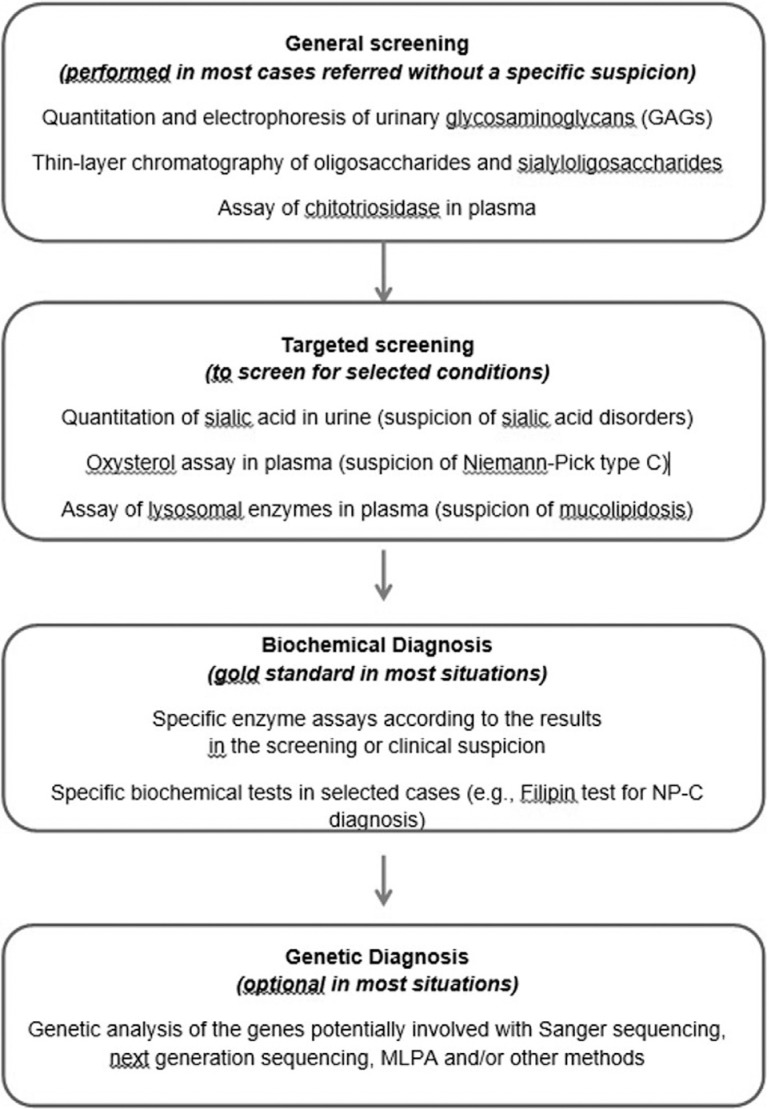
Flowchart of the investigation for LSDs.

Patient and laboratory records from individuals who had been diagnosed with an LSD at MGS/HCPA from 1982 to 2015 were analyzed. For some cases, the LSD investigation was initiated by urine screening, with quantitation and electrophoresis of urinary glycosaminoglycans (GAGs). Thin-layer chromatography of oligosaccharides and sialyloligosaccharides, chitotriosidase assays in plasma, and other selected procedures were performed according to clinical suspicion. The diagnoses were usually confirmed by specific fluorimetric, colorimetric or radioisotopic enzyme assays and/or by identification of pathogenic mutations, typically from blood samples (dried blood spots-DBS, plasma, leukocytes), and, when necessary, using fibroblasts cultivated from skin biopsies. When only DBS were available, enzyme assays were considered diagnostic when performed at least twice (in two independent samples). When necessary (mainly during the earlier years of the study period), the samples were sent to reference laboratories in other countries for complementary analyses. A laboratory workflow chart is shown in Figure 1, and the enzyme assays performed in the laboratory are listed in Table 1.

**Table 1:** Specific enzyme assays performed for the diagnosis of LSDs in MGS/HCPA.

Disease	Enzyme	Sample*
Acid lipase deficiency	Lysosomal Acid lipase	L, F, DBS
Fabry disease	α-Galactosidase A	P, L, F, DBS
Farber disease	Ceramidase	F
Fucosidosis	α-Fucosidase	L, F
Gaucher disease	β-Glucosidase	L, F, DBS
GM1-gangliosidosis/	β-Galactosidase	L, F, DBS
Galactosialidosis/MPS IVB		
GM2-gangliosidosis	Hexosaminidases	P, L, F, DBS
Tay-Sachs/Sandhoff		
GM2-gangliosidosis B1 variant	Hexosaminidase A (MUGS)	P, L, F, DBS
Krabbe disease	Galactocerebrosidase	L, F
α-Mannosidosis	α-Mannosidase	L, F, DBS
β-Mannosidosis	β-Mannosidase	L, F
Metachromatic leukodystrophy	Arylsulfatase A	L, F
MPS I	α-Iduronidase	P, L, F, DBS
MPS II	Iduronate sulfatase	P, L, F, DBS
MPS IIIA	Heparan sulfamidase	L, F
MPS IIIB	N-acetyl-α- glucosaminidase	P, L, F, DBS
MPS IIIC	Acetyl-CoA-glucosaminide-N-acetyltransferase	L, F
MPS IIID	N-acetyl-glucosamine-6-sulfatase	L, F
MPS IVA	N-acetylgalactosamine-6-sulfatase	L, F, DBS
MPS IVB	β-Galactosidase	L, F, DBS
MPS VI	Arylsulfatase B	L, F, DBS
MPS VII	β-Glucuronidase	P, L, F, DBS
Neuronal ceroid lipofuscinosis (CLN1)	Palmitoyl	L, F, DBS
	protein thioesterase	
Neuronal ceroid lipofuscinosis (CLN2)	Tripeptidyl peptidase	L, F, DBS
Niemann-Pick disease A/B	Sphingomyelinase	L, F, DBS
Pompe disease	α-Glucosidase	L, F
Schindler disease	N-acetylgalactosaminidase	P, L, F, DBS
Sialidosis/Galactosialidosis	Neuraminidase	F

* P: plasma; L: leukocytes; F: fibroblasts; DBS: dried blood spot samples.

Since 1988, prenatal diagnosis has been offered for those families for which a previous LSD diagnosis was well established in an index case, or for previously identified heterozygous couples (or for women carrying mutations for an X-linked disorder).

## Results

**Figure 2:**
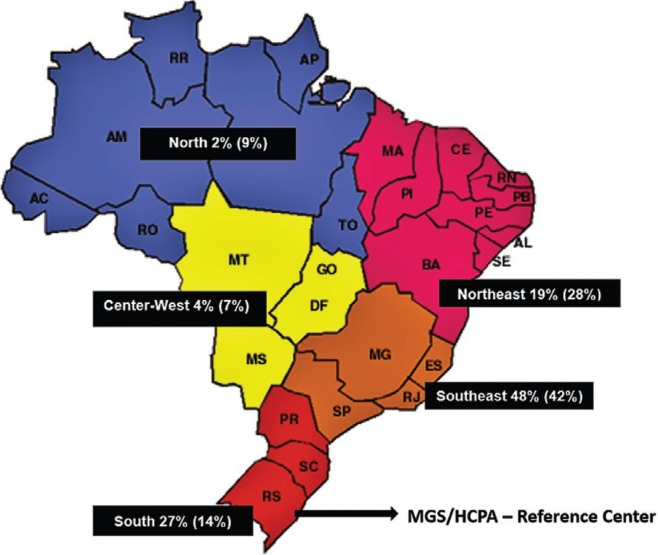
Percentage of LSDs diagnoses from different Brazilian regions (percentage of the Brazilian population living the region indicated between parenthesis).

From 1982 to 2015, 72,797 high-risk patients were investigated for IEM, as referred by several services from different regions of Brazil, other countries in Latin America, and occasionally Africa or Asia. During this period, an IEM diagnosis was confirmed in 4,489 (6.44% of all patients investigated) cases, and of these patients, 3,211 had LSDs (71.6% of the IEM cases and 4.41% of all patients investigated). From these 3,211 cases for which an LSD diagnosis was confirmed, 3,099 were from Brazil. The number of cases diagnosed according to the Brazilian state of origin is shown in Table 2, and the distribution of these diagnoses, including the percentage of the Brazilian population living in each region, is shown in Figure 2. The origins of samples sent from foreign countries are shown in Table 3.

**Table 2:** Number of cases diagnosed from each Brazilian state, considering the 3,038 patients for whom this information was available.

Region/States	Number of patients diagnosed with LSD
South Region	
Paraná	172
Santa Catarina	82
Rio Grande do Sul	539
Total	793
Southeast Region	
Minas Gerais	248
São Paulo	934
Rio de Janeiro	246
Espirito Santo	52
Total	1480
Centerwest Region	
Mato Grosso	2
Goias	50
Distrito Federal	76
Mato Grosso do Sul	1
Total	129
Northeast Region	
Maranhão	29
Ceará	105
Piauí	17
Rio Grande do Norte	31
Paraíba	55
Pernambuco	157
Alagoas	39
Sergipe	0
Bahia	139
Total	572
North Region	
Acre	3
Rondônia	0
Amazonas	25
Pará	36
Roraima	0
Amapá	0
Total	64
TOTAL	3038

**Table 3:** Number of LSD diagnoses in patients from foreign countries*.

Region	Number of patients
*Latin America*	*91*
Argentina	9
Bolivia	1
Chile	39
Colombia	4
Cuba	3
Mexico	5
Nicaragua	1
Panama	2
Paraguay	2
Peru	16
Uruguay	9
*Other*	*21*
Algeria	3
Iran	3
Libya	1
Saudi Arabia	13
United Arab Emirates	1
Total	112

* For most diagnoses, DBS samples were used and the result was confirmed in a second DBS sample in most cases (in just a few foreign cases it was possible to obtain a viable blood sample to perform the confirmation in leukocytes).

Considering only the 3,099 confirmed Brazilian cases, the most common LSDs diagnosed (over 50 cases each) were Gaucher disease (725 cases), MPS II (343 cases), MPS VI (238 cases), MPS I (225 cases), acid sphingomyelinase deficiency/ASMD (199 cases), MPS IVA (153 cases), GM1 gangliosidosis (176 cases), Niemann-Pick C (150 cases), Metachromatic Leukodystrophy (150 cases), Tay-Sachs disease (122 cases), Fabry disease (104 cases), MPS IIIB (88 cases), Krabbe disease (96 cases), MPS IIIA (53 cases), and MPS IIIC (52 cases). It is interesting to note that of all patients diagnosed with Tay-Sachs disease, 44% were of the B1 variant. The number of diagnosed cases for each LSD from the 1982-2015 period is shown in Table 4.

**Table 4:** Lysosomal storage diseases diagnosed from 1982 to 2015 in Brazilian patients*.

Lysosomal storage disease	Number of confirmed diagnoses	Additional probable diagnosis**
Mucopolysaccharidoses		
Mucopolysaccharidosis type I	225	11
Mucopolysaccharidosis type II	343	4
Mucopolysaccharidosis type IIIA	52	-
Mucopolysaccharidosis type IIIB	88	-
Mucopolysaccharidosis type IIIC	52	-
Mucopolysaccharidosis type IVA	153	-
Mucopolysaccharidosis type IVB	13	-
Mucopolysaccharidosis type VI	238	3
Mucopolysaccharidosis type VII	20	-
Multiple sulphatase deficiency	6	-
Glycoproteinoses		
Aspartylglucosaminuria	1	-
Fucosidosis	4	-
Galactosialidosis	19	-
α-Mannosidosis	7	1
Mucolipidosis II/III	41	8
Sialidosis	14	-
Sphingolipidoses		
Fabry disease	104	3
Gaucher disease	725	2
GM1 gangliosidosis	175	-
GM2 Tay-Sachs disease (44% B1)	121	3
GM2 Sandhoff disease	28	-
Krabbe disease	96	-
Metachromatic Leukodystrophy	150	-
Niemann-Pick type A/B disease	199	5
		
Other LSDs		
Lysosomal acid lipase deficiency	10	7
Neuronal Ceroid lipofuscinosis 1 (CLN1)	3	-
Neuronal Ceroid lipofuscinosis 2 (CLN2)	14	3
Niemann-Pick type C	150	-
Pompe disease	47	9
Salla disease	1	-
Total	3099	59

* Classified as proposed by Kingma *et al*., 2015).

** Cases with only one sample of DBS analyzed, not considered as confirmed cases.

One-hundred twenty cases were evaluated by prenatal diagnosis since 1988, and a positive result was found in 34 pregnancies (28.3%). The majority of prenatal diagnoses were in pregnancies at risk for GM1 gangliosidosis (37 cases), Mucopolysaccharidosis type I (16 cases), Tay-Sachs diseases (14 cases), Mucopolysaccharidosis type II (12 cases) or Metachromatic Leukodystrophy (10 cases).

**Figure 3:**
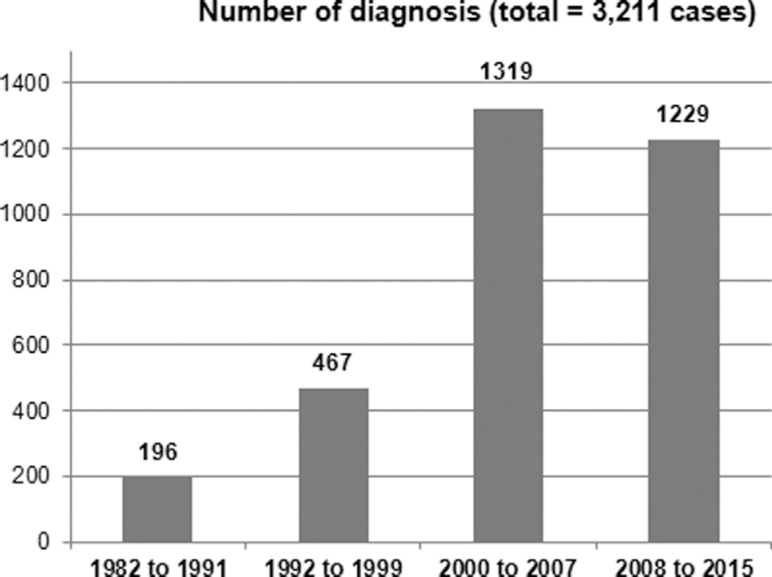
Number of lysosomal storage diagnosis by period (1982 to 2015).

Figure 3 shows the number of diagnosed patients by time period, divided as follows: 1982/1991 - implementation of diagnostic methods, few enzyme assays performed; 1992/1999 - growing number of enzyme assays performed in the laboratory; 2000/2007 - introduction of enzyme assays using dried blood spots, establishment of the MPS Brazil Network to facilitate diagnosis; and 2008/2015 - new screening protocols for LSDs in high-risk patients, implementation of the LSD Brazil network. The number of diagnoses increased significantly when we compared the first ten years (196 cases) with the next 24 years (3,019 cases) of the study period. In the last 16 years, approximately 160 new cases were identified per year.

Considering the period from 2000 to 2013 (for which the number of live births is available in the Brazilian Health System Database), 2,092 patients were diagnosed with an LSD. During this period, 41,719,041 live births occurred with 1 case of LSD per 19,942 live births. The minimal frequency estimated for each LSD in Brazil is presented in Table 5.

**Table 5:** Minimal frequency of LSDs in Brazil and comparison with data from other countries.

		Brazil^1^	Australia^2^	The Netherlands^3^	British Columbia^3^	Portugal^3^	Czech Republic^3^	Eastern Province of Saudi Arabia^2^	United Arab Emirates^3^
Disease	*Clinical phenotype*	*Present study*	*Meikle et al., 1999*	*Poorthuis et al., 1999*	*Applegarth et al., 2000*	*Pinto et al., 2004*	*Poupetova et al., 2010*	*Moammar et al., 2010*	*Al-Jasmi et al., 2013*
α-N-Acetylgalactosaminidase deficiency	Schindler disease; Kanzaki disease			0.20			0		0
Acid lipase deficiency	Cholesterol ester storage disease; Wolman disease	0.011	0.19		0.58		0.27	1.0	0
Aspartylglucosaminuria		0	0.05	0.13		1.72			0
Cystinosis			0.52		0.68			1.0	0.25
Danon disease									
Fabry disease	Fabry disease	0.22	0.85	0.21	0.29	0.12	0.52	5.0	0.25
Farber lipogranulomatosis	Farber disease								0.96
Fucosidosis		0.004		0.05		0	0		2.02
Galactosialidosis types I/II		0.02		0.04	0.39	0.77	0	1.0	0
Gaucher disease	Gaucher disease	1.43	1.75	1.16	0.39	1.35	1.13		0.25
Globoid cell leukodystrophy	Krabbe disease	0.14	0.71	1.35	0.29	1.21	0.4		0
Glycogen storage disease II	Pompe disease	0.10	0.68	2.00	0.87	0.7	0.37		2.66
GM1-gangliosidosis types I/II/III		0.18	0.26	0.41	0.19	0.62	0.26	2.0	4.66
GM2-gangliosidosis type AB									
GM2-gangliosidosis type I (B variant)	Tay-Sachs disease	0.21	0.50	0.41	0.29	3.13	0.3		0.74
GM2-gangliosidosis type II (O variant)	Sandhoff disease	0.04	0.26	0.34	0.19	1.49	0.19	5.0	1.21
α-Mannosidosis		0.016	0.09	0.09		0.12	0.38	1.0	1.51
β-Mannosidosis				0.13		0.12	0.16		0
Metachromatic leukodystrophy	0.21		1.09	1.42	0.58	1.85	0.69		1.5
Mucolipidosis type I	Sialidosis types I/II								
Mucolipidosis types II/III	I-cell disease; pseudo-Hurler polydystrophy	0.06	0.31	0.24	0.29	2.7	0.22		1.35
Mucolipidosis type IIIC	pseudo-Hurler polydystrophy								
Mucolipidosis type IV									
MPS I	Hurler; Hurler-Scheie; Scheie syndrome	0.31	1.14	1.19	0.58	1.33	0.72	4.0	0.25
MPS II	Hunter Syndrome	0.71	0.74	0.65	0.10	1.09	0.43		0
MPS IIIA	Sanfilippo syndrome	0.08	0.88	1.16	0.29	0	0.47		0
MPS IIIB	Sanfilippo syndrome	0.12	0.47	0.42		0.72	0.02		1.05
MPS IIIC	Sanfilippo syndrome	0.09	0.07	0.21		0.12	0.42		
MPS IIID	Sanfilippo syndrome	0	0.09	0.10			0		0
MPS III (all types)	Sanfilippo syndrome	0.29						2.0	
MPS IVA	Morquio syndrome	0.21	0.59	0.22	0.48	0.6	0.71		1.41
MPS IVB	Morquio syndrome	0.016		0.14			0.02		
MPS IV (both types)	Morquio syndrome	0.22						4.0	
MPS VI	Maroteaux-Lamy syndrome	0.37	0.43	0.15	0.48	0.42	0.05	8.0	2.51
MPS VII	Sly syndrome	0.026	0.05	0.24	0.29	0	0.02		
MPS IX		0							
Multiple sulphatase deficiency		0.011	0.07	0.05	0.10	0.48	0.26		0
Neuronal Ceroid Lipofuscinosis 1 (CLN1)	Santavuori disease	0.0024							
Neuronal Ceroid Lipofuscinosis 2 (CLN2)	Jansky-Bielschowsky disease	0.02							
Neuronal Ceroid Lipofuscinosis 3 (CLN3)	Batten disease							5.0	
Neuronal Ceroid Lipofuscinosis 5 (CLN5)	Finnish variant late-infantile neuronal ceroid lipofuscinosis								
Neuronal Ceroid Lipofuscinosis 6 (CLN6)	Variant late-infantile neuronal ceroid lipofuscinosis								
Neuronal Ceroid Lipofuscinosis 8 (CLN8)	Northern epilepsy								
Niemann-Pick type A/B	Niemann-Pick disease	0.33	0.40	0.53		0.6	0.33	5.0	0.25
Niemann-Pick type C	Niemann-Pick disease	0.304	0.47	0.35		2.2	0.91	1.0	0.25
Prosaposin deficiency	Atypical Gaucher disease								
Pycnodysostosis									
Sialic acid storage disease	Infantile free sialic acid storage disease; Salla disease	0.0024	0.19	0.07	0.19		0.02		0
Sialidosis		0.02	0.02	0.05		0	0.07		0
Sialuria									

1 Total number of cases diagnosed from 2000 to 2013 (14 years) divided by the total number of births in the same period.

2 Total number of diagnosed cases within a certain period of time divided by the total number of births in the same period.

3 Total number of diagnosed cases born within a certain period of time divided by the total number of births in the same period.

## Discussion

As there are only few other laboratories investigating selected LSDs in Brazil, it is not possible to say that the results presented in this report represent the overall data for LSDs in Brazil. However, as shown in Table 2 and Figure 2, it is clear that the reference laboratory covers the whole country, as cases were identified in all Brazilian regions. The cases were nearly evenly distributed among the Brazilian regions, with a relative overrepresentation of the South and Southeast (possibly due to the location of the reference laboratory and the increased availability of better health system facilities in these regions) and a relative underrepresentation of the Northeast, Center-West and North (possibly due to health system deficiencies and/or logistics difficulties in sending samples to the reference laboratory).

The continuous introduction of diagnostic methods during the study period, such as specific enzyme assays, may explain the increased diagnostic rates during the later years. Additionally, the incorporation of enzyme assays performed using DBS in the first years of this century simplified the collection and shipment of samples and may have also played a role in increasing LSD diagnoses, even in services located in distant regions and/or in foreign countries. Additionally, some enzyme assays were introduced only more recently, such as for instance the lysosomal acid lipase assay, which has only been available since 2012.

In a previous study published by our group, Gaucher disease and GM1 gangliosidosis were the conditions with the highest incidence in our population (Coelho *et al*., 2001). At the time that study was published, no specific treatment for MPS was available. In the present investigation, we can see that MPS I, MPS II and MPS VI are also among the most frequent LSDs diagnosed. Similar results have also been observed in Colombia, where this group of diseases, mainly represented by MPS, is more frequently reported (Barrera and Uribe, 1994). This finding may reflect a higher awareness of referring health professionals on diseases that have specific treatment available.

LSDs were detected in 4.41% of all samples referred for IEM investigation, representing the most frequent IEM group in our service (71.6% of IEM diagnosis). It is important to emphasize that our laboratory was the first to offer specific diagnosis of LSD in Brazil, and is also the most complete LSD laboratory in the country. It soon became recognized as a specialized center for these conditions, which may explain the high proportion of this group of disorders among the cases of diagnosed IEM. We have to highlight that LSDs are clinically more evident to physicians due to their phenotypic appearance, and are, thus, more promptly suspected than other IEM.

Due to the rarity of the LSDs and the relatively sophisticated methods required for their diagnosis, the identification of these conditions represents a challenge to clinicians, especially in developing countries. Aiming to improve the access of families and health professionals to information and diagnosis of LSDs, an innovative project was set up in Brazil, initially for MPS and later for other LSDs. In 2004, a partnership among Brazilian medical services that addresses MPS patients was created, the MPS Brazil Network, with headquarters at MGS/HCPA. Since then, the network provides a wide range of information about MPS, performs the laboratory tests necessary for the diagnosis, and organizes regular meetings in order to keep families updated with the most recent advances in the field. The network’s objective is not only to provide the tests for diagnosis, but also to support research, courses, workshops and training for other services interested in MPS to support earlier diagnosis of these conditions. This initiative is supported with public and private grants, which enable it to provide the services free of charge to the requesting physician, making information and diagnostic tests available even for families that usually do not have access to sophisticated healthcare facilities (Giugliani, 2012b). Since the MPS Brazil Network initiated its activities, the average number of patients diagnosed with MPS by year has doubled. The success of this template in MPS diagnosis has stimulated the creation of a similar network for Niemann-Pick type C (the NPC Brazil Network) and for other lysosomal diseases (the LSD Brazil Network). This model was probably a leading factor for the significant increase in the number of cases diagnosed in the 2000-2007 and 2008-2015 periods compared to the diagnoses made in the 1982-1991 and 1991-1999 periods, as shown in Figure 3.

These results indicate that LSDs, although individually rare, may be frequent when the investigation is concentrated in reference laboratories, with 1 out of 22 patients identified with an LSD among the cases referred for suspicion of an inherited metabolic disease. The relative frequency of LSDs, as shown in Table 3, provides a useful guideline for health authorities to plan the care of these patients because there are specific therapies available for many of the most frequent conditions. Although the minimal frequency for each LSD displayed in Table 5 may represent an underestimate (as many cases are still undiagnosed and include data from only one of the diagnostic centers), this is the first attempt to make this estimate for Brazil. A large number of cases enables centers to obtain experience in managing these conditions, to perform natural history studies, and to participate in clinical trials. It is important to mention that the majority of patients that were identified could benefit from the therapeutic alternatives that are already available for these conditions, or from those which are being developed.
